# Relative time-intensity curve: a new method to differentiate benign and malignant lesions on breast MRI

**DOI:** 10.1186/1470-7330-14-S1-P26

**Published:** 2014-10-09

**Authors:** H Sherif, A Mahfouz, A Kambal, A Sayedin, I Mujeeb

**Affiliations:** 1 Hamad Medical Corporation, Doha, Qatar

## Purpose

Benign/malignant overlap exists on time-intensity curve (TIC) of dynamic gadolinium-enhanced breast MRI. This study presents a new method for TIC generation to decrease overlap and improve accuracy.

## Material and methods

MR images of 100 patients with enhancing breast lesions (64 malignant, 36 benign) obtained before and repeatedly after intravenous injection of Gd-DOTA were evaluated. Signal intensity of lesions (SIlesion) and breast (SIbreast) were measured and TIC obtained. Relative signal intensity of the lesion was calculated as (SIlesion-SIbreast)/SIbreast and plotted versus time to obtain relative TIC. Four parameters were evaluated for diagnosis of carcinoma: peak enhancement (PE), initial enhancement slope (S), time-to-peak (TTP), and washout ratio (WO). Comparison of parameter performance on TIC and relative TIC has been done by the Student’s T test and the Receiver-Operator Curve (ROC) analysis.

## Results

On TIC, TTP has been the only discriminating factor. When threshold for carcinoma has been set at TTP≤2 min, sensitivity, specificity, positive predictive value, negative predictive value, and accuracy have been 36, 100, 100, 43, and 57% respectively. On relative TIC, accuracy of TTP has increased to 89%. Area under the ROC curve for TTP has improved from 0.77 for TIC to 0.86 for relative TIC (p<0.05). WO ratio has become a second discriminating factor on relative TIC with more washout in malignant lesions (WO=41±32) than benign lesions (WO= 11±19) (p <0.05). PE and S were not statistically significant on TIC or relative TIC.

## Conclusion

The use of relative TIC improves the discrimination of benign and malignant breast lesions.

